# New Flavonolignan Glycosides from the Aerial Parts of *Zizania latifolia*

**DOI:** 10.3390/molecules20045616

**Published:** 2015-03-30

**Authors:** Seung-Su Lee, Nam-In Baek, Yoon-Su Baek, Dae-Kyun Chung, Myoung-Chong Song, Myun-Ho Bang

**Affiliations:** 1 Graduate School of Biotechnology and Research Institutee of life Science & Resources, Kyung Hee University, Yongin-si, Gyeonggi-do 446-701, Korea; E-Mails: ldkhhghh@khu.ac.kr (S.-S.L.); nibaek@khu.ac.kr (N.-I.B.); dkchung@khu.ac.kr (D.-K.C.); 2 Skin Biotechnology Center, Kyung Hee University, Suwon 433-766, Korea; 3 Department of Oriental Medicinal Material and Processing, College of Life Science, Kyung Hee University, Yongin-si, Gyeonggi-do 446-701, Korea; E-Mail: acplant@khu.ac.kr; 4 Department of Chemistry and Nano Science, Ewha Womans University, Seoul 120-750, Korea; E-Mail: smch517@hanmail.net

**Keywords:** *Zizania latifolia*, flavonolignan glycoside, tricin-7-*O*-β-d-glucopyranose

## Abstract

Two new flavonolignan glycosides, tricin-4'-*O*-(*threo*-β-guaiacylglyceryl) ether 7''-*O*-β-d-glucopyranose (**4**) and tricin-4'-*O*-(*erythro*-β-guaiacylglyceryl) ether 7''-*O*-β-d-glucopyranose (**5**) were isolated from the roots of *Zizania latifolia*, together with tricin-7-*O*-β-d-glucopyranose (**1**), tricin-4'-*O*-(*threo*-β-guaiacylglyceryl) ether 7-*O*-β-d-glucopyranose (**2**), and tricin-4'-*O*-(*erythro*-β-guaiacylglyceryl) ether 7-*O*-β-d-glucopyranose (**3**). Their structures were identified on the basis of spectroscopic techniques, including HR-ESI/MS, 1D-NMR (^1^H, ^13^C, DEPT), 2D-NMR (gCOSY, gHSQC, gHMBC), and IR spectroscopy.

## 1. Introduction

**Figure 1:**
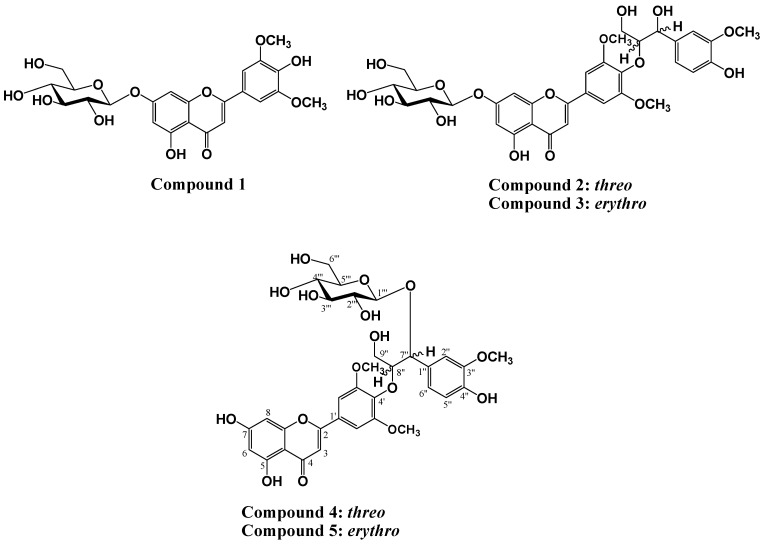
Chemical structures of isolated compounds **1**–**5**.

*Zizania latifolia* Turcz (wild rice) is cultivated in various regions of Southeastern Asia, including Korea, Japan, and China. Particularly, in China, wild rice grain is used as an important traditional medicine to treat anemia and fever in addition to heart, kidney, and liver disorders. It has been reported that wild rice species possess rich nutrient content [1,2], as well as diverse biological activities such as anti-obesity [3], anti-oxidant [4], anti-inflammatory and anti-allergenic effects [5]. Previous phytochemical studies on *Z. latifolia* have confirmed the presence of flavonolignan groups along with tricin. In order to identify phytochemical components of *Z. latifolia*, isolation and structural determination of two new flavonolignan glycosides, tricin-4'-*O*-(*threo*-β-guaiacylglyceryl) ether 7''-*O*-β-d-glucopyranose (**4**) and tricin-4'-*O*-(*erythro*-β-guaiacylglyceryl) ether 7''-*O*-β-d-glucopyranose (**5**), a flavone glycoside, tricin-7-*O*-β-d-glucopyranose (**1**), two known flavonolignan glycosides tricin-4'-*O*-(*threo*-β-guaiacylglyceryl) ether 7-*O*-β-d-glucopyranose (**2**), and tricin-4'-*O*-(*erythro*-β-guaiacylglyceryl) ether 7-*O*-β-d-glucopyranose (**3**) were performed. These compounds are of particular interest since flavonolignan glycosides have been reported for the first time from *Z. latifolia* (Figure 1).

## 2. Results and Discussion

The aerial parts were extracted using 80% MeOH, and the concentrated extracts were successively partitioned using EtOAc, n-BuOH, and H_2_O. Repeated SiO_2_, and ODS-A column chromatographic separation of the EtOAc fraction produced a purified flavone glycoside, and four flavonolignans including two new flavonolignan glycosides. These compounds were identified as tricin-7-*O*-β-d-glucopyranose (**1**), tricin-4'-*O*-(*threo*-β-guaiacylglyceryl) ether 7-*O*-β-d-glucopyranose (**2**), tricin-4'-*O*-(*erythro*-β-guaiacylglyceryl) ether 7-*O*-β-d-glucopyranose (**3**), tricin-4'-*O*-(*threo*-β-guaiacylglyceryl) ether 7''-*O*-β-d-glucopyranose (**4**) and tricin-4'-*O*-(*erythro*-β-guaiacylglyceryl) ether 7''-*O*-β-d-gluco-pyranose (**5**), respectively, based on interpretation of the spectroscopic data including NMR, MS, and IR spectrascopy, and confirmed by comparison of the data with those reported in the literature [6,7,8]. All compounds were isolated for the first time from *Z. latifolia*.

**Figure 2:**
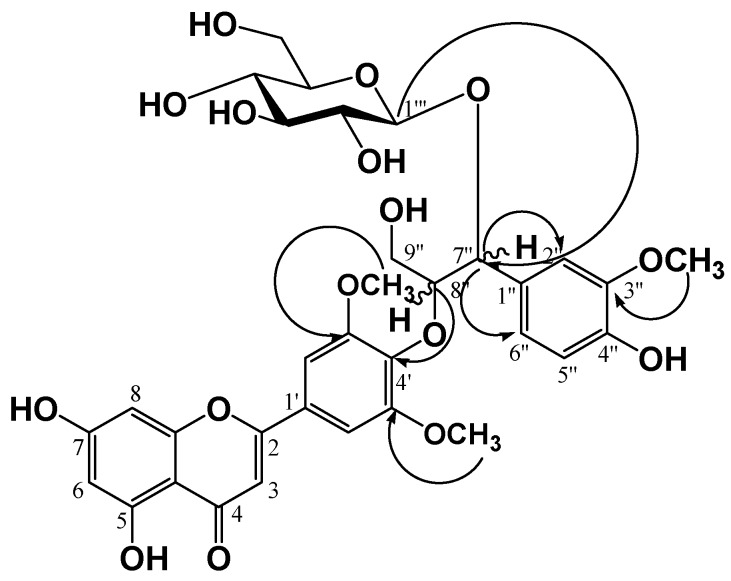
Key HMBC correlations of compound **4**.

Compound **4** (a yellow amorphous powder) was developed by TLC, followed by spraying with 10% H_2_SO_4_ and heating to produce a dark yellow color. In the ^1^H-NMR spectrum, three olefin methine proton signals at δ 7.50 (1H, d, *J* = 1.6 Hz, H-2''), 7.37 (1H, dd, *J* = 8.0, 1.6 Hz, H-6''), and 7.18 (1H, d, *J* = 8.0 Hz, H-5'') were observed due to a 1,2,4-trisubstituted benzene ring along with five olefine methine proton signals at δ 7.27 (2H, br. s, H-2', 6'), 6.97 (1H, s, H-3), 6.83 (1H, d, *J* = 2.0 Hz, H-8), and 6.72 (1H, d, *J* = 2.0 Hz, H-6) due to a flavone moiety. In the oxygenated region, two oxygenated methylene proton signals at δ 5.89 (1H, d, *J* = 6.0 Hz, H-7'') and 5.19 (1H, m, H-8''), an oxygenated methylene proton signal at δ 4.75 (1H, dd, *J* = 12.4, 3.6 Hz, H-9''a) and 4.36 (1H, dd, *J* = 12.0, 2.8 Hz, H-9''b), and three methoxy proton signals at δ 3.81 (6H, s, H-3', 5'-OCH_3_) and 3.74 (3H,s, H-3''-OCH_3_) were observed. There were also oxygenated methine and methylene signals for a monosaccharide moiety observed in the region from δ 4.12 to 4.28, including one hemiacetal proton signal at δ 5.54 (1H, d, *J* = 8.0 Hz, H-1''') with a ^3^*J*_H1''/H2''_ coupling constant of 8.0 Hz, indicating an anomer hydroxy in β-configuration. The relative configuration of chiral carbons, C-7'' and C-8'', in compound (**4**) was deduced to be *threo* type from the coupling constant (*J* = 6.0 Hz) between the oxygenated methine signals H-7'' (δ_H_ 5.89) and H-8'' (δ_H_ 5.19). It was reported that the J_H_-7'',8'' coupling constant of proton resonance in the guaiacylglyceryl moiety can be used to distinguish between the *erythro* and *threo* forms, with *J* values of 4.0–5.0 and 6.0–7.0 Hz, respectively [9,10,11]. The ^13^C-NMR spectrum showed 33 carbon signals including three methoxy carbons [δ 52.3 (C-3', 5'-OCH_3_) and 56.6 (C-3''-OCH_3_)], confirming that compound **4** was a flavonoid with a phenylpropanoid moiety and hexose moiety. In the low-magnetic field region, a conjugated ketone carbon δ 183.9 (C-4), seven oxygenated olefine quaternary carbons [δ 167.3 (C-7), 165.0 (C-2), 164.3 (C-5), 159.7 (C-9), 155.1 (C-3', 5'), 141.3 (C-4')], two olefine quaternary carbons [δ 127.9 (C-1'), 106.3 (C-10)], and five olefin methine carbons [δ 107.0 (C-3), 105.9 (C-2', 6'), 101.3 (C-6), 96.3 (C-8)] signals were observed, indicating the presence of a flavone moiety. In addition, two oxygenated olefine quaternary carbons [δ 149.8 (C-3''), 149.1 (C-4'')], one olefine quaternary carbon δ 133.1 (C-1''), and three olefine methine carbons [δ 122.8 (C-6''), 117.0 (C-5''), 114.0 (C-2'')], two oxygenated methine carbons [δ 87.8 (C-8''), 82.6 (C-7'')] and one oxygenated methylene carbon δ 62.7 (C-9'') signals were observed, indicating the presence of a phenylpropanoid moiety. In the HMBC spectrum, the oxygenated methine proton δ_H_ 5.19 (1H, m, H-8'') showed a correlated cross peak with an oxygenated olefine quaternary carbon δ_C_ 141.3 (C-4′), indicating that the flavone and phenylpropanoid moieties were linked between δ_C_ C-8'' and δ_C_ C-4' via an ether linkage, as well as a cross-peak between an anomer proton signal at δ_H_ 5.54 (1H, d, *J* = 8.0 Hz, H-1''') and an oxygenated methine carbon signal at δ_C_ 82.6 (C-7''), indicating that glucose was attached to the C-7'' of the phenylpropanoid through a glycosidic likage (Figure 2). Finally, the structure of compound (**4**) was determined to be tricin-4'-*O*-(*threo*-β-guaiacylglyceryl) ether 7''-*O*-β-d-gluco-pyranose, which is salcolin A 7''-*O*-β-d-glucopyranose. The molecular weight was determined to be 688.2003 from the pseudomolecular ion peak at *m/z* 687.1962 [M−H]^−^ in the negative high resolution ESI-MS (calculated for C_33_H_35_O_16_).

Signals in the ^1^H-NMR (400 MHz, pyridine-*d*_5_, δ_H_) and ^13^C-NMR (100 MHz, pyridine-*d*_5_, δ_C_) spectra of compound **5** were almost the same as those of compound **4**, with the exception of the relative stereostructure of C-7'' and C-8''. Compound **5** was confirmed to be *erythro* type from the coupling constant between the oxygenated methine signals of H-7'' and H-8'' (*J* = 4.8 Hz). The ether linkage (C-8''/C-4') and glycosidic likage (C-1'''/C-7'') were confirmed from the cross peak between the oxygenated methine proton δ_H_ 5.16 (1H, m, H-8'') and oxygenated olefine quaternary carbon δ_C_ 142.0 (C-4'), as well as the cross-peak between the anomer proton signal at δ_H_ 5.35 (1H, d, *J* = 8.0 Hz, H-1''') and a oxygenated methine carbon signal at δ_C_ 81.9 (C-7'') in the HMBC spectrum. Consequently, the structure of compound **5** was determined to be tricin-4'-*O*-(*erythro*-β-guaiacylglyceryl) ether 7''-*O*-β-d-glucopyranose, which is salcolin B 7''-*O*-β-d-glucopyranose. The molecular weight was determined to be 688.2003 from the pseudomolecular ion peak at *m/z* 687.1952 [M−H]^−^ in the negative high resolution ESI-MS (calculated for C_33_H_35_O_16_).

## 3. Experimental

### 3.1. General

Preparative-liquid chromatography (prep-LC) (LC-Forte/R, YMC, Kyoto, Japan) was carried out using cartridge YMC-DispoPack (ODS-25, 80 g, and 120 g, YMC). Silica gel (230–400 mesh, Merck, Darmastdt, Germany) and Sephadex LH-20 (Merck) were used for column chromatography. Thin–layer Chromatography (TLC) analysis was performed using on a Kiesel gel 60F_254_ plate (Merck) and a RP-18 F_254s_ plates (Merck), and detection was performed by a UV lamp at 254 and 365 nm and 10% H_2_SO_4_ solution by spraying and heating. ^1^H and ^13^C-NMR along with 2D-NMR data were obtained on a Bruker AVANCE II 400 (^1^H-NMR at 400 MHz, ^13^C-NMR at 100 MHz) spectrometer (Bruker, Rheinstetten, Germany) in pyridine-*d*_5_ with TMS as internal standard. ESI/MS was obtained on a AB SCIEX triple TOF 5600 mass spectrometer (AB SCIEX, Concord, ON, Canada). All the reagent grade chemicals were purchased from Sigma Chemical Co. (St. Louis, MO, USA).

### 3.2. Plant Material

*Plant sample*. Dried aerial parts of *Z. latifolia* was purchased from store located at Yeongcheon City, Gyeongbuk, Korea. A voucher specimen (KHU-0130301) was deposited at the Laboratory of Natural Products Chemistry, Kyung Hee University, Yongin, Korea.

### 3.3. Extraction and Isolation

Dried powder (5 kg) was extracted at room temperature with 80% MeOH (45 L). The concentrated extract was partitioned with water (3 L), EtOAc (3 L) and n-BuOH (3 L) successively to produce. EtOAc extract (97 g, ZLE), n-BuOH extract (240 g, ZLB) and H_2_O extract (57 g, ZLW). The EtOAc extract was applied to a silica gel c.c. (12 × 14 cm), and eluted with CHCl_3_ : MeOH (50:1 → 30:1 → 20:1 → 12:1 → 10:1 → 7:1 → 5:1 → 3:1 → 1:1, each 3 L) monitoring by thin layer chromatography (TLC) to produce 13 fractions (ZLE-1 to ZLE-13). Fraction ZLE-12 (5.0 g) was combined and subjected to MPLC column chromatography: YMC-DispoPack AT (ODS-25:120 g, particle size: 25 μm). The fractions were analyzed by a UV detector at 254 and 356 nm, and the mobile phase consisted of A: 0.1% formic acid in water, B: 0.1% formic acid in acetonitrile with a flow rate of 10 mL/min. The gradient consisted of 10% B held for 20 min to 30% B at a rate of 0.25%/min, from 30% B to 50% B at a rate of 0.20%/min, from 50% B to 70% B at a rate of 0.25%/min, and from 70% B to 90% B at a rate of 0.30%/min. A total 11 fractions (ZLE-12-1 to ZLE-12-15) were obtained. ZLE-12-10 (642 mg) was subjected to MPLC column chromatography: YMC-DispoPack AT (ODS-25: 80 g, particle size: 25 μm), with A: 0.1% formic acid in water, B: 0.1% formic acid in acetonitrile as the mobile phase at a flow rate of 5 mL/min. The detector was maintained at 254 and 356 nm. The gradient consisted of 20% B to 50% B at a rate of 0.5%/min, and 50% B to 70% B at a rate of 0.6%/min. Compound **1** (32 mg, RT = 6.05 min), compound **2** (35 mg, RT = 7.8 min), compound **3** (42 mg, RT = 9.2 min), compound **4** (22 mg, RT = 12.4 min) and compound **5** (31 mg, RT = 14.6 min) were obtained.

### 3.4. Spectroscopic Data

*Tricin-7-O-β-d-glucopyranose* (**1**). Pale yellow powder (CH_3_OH); negative ESI/MS *m*/*z* 491 [M−H]^−^; IR (KBr) 3380, 2921, 1730, 1652, 1613, 1491,1358, 1084 cm^−1^; ^1^H-NMR (pyridine-*d_5_*) δ 7.55 (2H, s, H-2', 6'), 7.17 (1H, d, *J* = 2.0 Hz, H-8), 7.02 (1H, s, H-3), 6.86 (1H, d, *J* = 2.0 Hz, H-6), 5.78 (1H, d, *J* = 7.2 Hz, H-1''), 4.55 (1H, dd, *J* = 10.4, 1.2 Hz, H-6'''a), 4.38-4.10 (5H, m, H-2''', H-3''', H-4''', H-5''', 6'''b-OCH_2_), 3.87 (6H, s, OCH_3_-3', 5'). ^13^C-NMR (pyridine-*d_5_*) δ 182.8 (C-4), 164.9 (C-2), 164.0 (C-7), 162.5 (C-5), 157.8 (C-9), 149.5 (C-3', 5'), 142.2 (C-4'), 121.0 (C-1'), 106.5 (C-10), 105.1 (C-2', 6'), 104.5 (C-3), 101.7 (C-1''), 100.6 (C-6), 95.5 (C-8), 79.2 (C-3''), 78.4 (C-5''), 74.7 (C-2''), 71.0 (C-4''), 62.2 (C-6''), 56.5 (C-3', 5'-OCH_3_).

*Tricin-4*'*-O-(threo-β-guaiacylglyceryl) ether* *7-O-β-d-glucopyranose* (**2**). Yellow amorphous powder (CH_3_OH); Negative ESI/MS *m*/*z* 687 [M−H]^−^; IR (KBr) 3369, 2938, 1652, 1611, 1588, 1512, 1495, 1356, 1263, 1159, 1124, 841 cm^−1^; ^1^H-NMR (pyridine-*d_5_*) δ 7.53 (1H, s, H-2''), 7.37 (1H, dd, *J* = 8.0, 1.6 Hz, H-6''), 7.31 (2H, s, H-2', 6'), 7.26 (1H, d, *J* = 8.0 Hz, H-5''), 7.14 (1H, d, *J* = 2.4 Hz, H-8), 7.01 (1H, s, H-3), 6.85 (1H, d, *J* = 2.0 Hz, H-6), 5.76 (1H, d, *J* = 7.2 Hz, H-1'''), 5.70 (1H, d, *J* = 5.2 Hz, H-7''), 5.08 (1H, q, *J* = 5.2, 3.2 Hz, H-8''), 4.65 (1H, dd, *J* = 12.0, 5.2 Hz, H-9''e), 4.54 (1H, dd, *J* = 12.0, 2.4 Hz, H-6'''a), 4.28 (1H, m, H-9''a), 4.36-4.27 (5H, m, H-2''', H-3''', H-4''', H-5''', 6'''b-OCH_2_), 3.84 (6H, s, OCH_3_-3', 5'), 3.74 (3H, s, OCH_3_-3''). ^13^C-NMR (pyridine-*d_5_*) δ 184.1 (C-4), 165.5 (C-7), 165.4 (C-2), 163.7 (C-5), 159.1 (C-9), 155.3 (C-3', 5'), 149.6 (C-3''), 148.6 (C-4''), 141.9 (C-4'), 135.6 (C-1''), 127.7 (C-1'), 121.8 (C-6''), 117.3 (C-5''), 112.9 (C-2''), 107.8 (C-10), 107.1 (C-3), 106.1 (C-2', 6'), 103.0 (C-1'''), 102.1 (C-6), 96.8 (C-8), 89.4 (C-8''), 80.5 (C-3''′), 79.7 (C-5'''), 76.0 (C-4'''), 75.1 (C-7''), 72.4 (C-2'''), 63.5 (C-6'''), 62.9 (C-9''), 57.7 (C-3', 5'-OCH_3_), 57.1 (C-3''-OCH_3_).

*Tricin-4*'*-O-(erythro-β-guaiacylglyceryl) ether* *7-O-β-d-glucopyranose* (**3**). Yellow amorphous powder (CH_3_OH); Negative ESI/MS *m*/*z* 687 [M−H]^−^; IR (KBr) 3364, 2933, 1649, 1607, 1590, 1495, 1457, 1358, 1121, 832 cm^−1^; ^1^H-NMR (pyridine-*d_5_*) δ 7.57 (1H, s, H-2''), 7.46 (1H, dd, *J* = 8.0, 1.2 Hz, H-6''), 7.29 (2H, s, H-2', 6'), 7.26 (1H, d, *J* = 8.0 Hz, H-5''), 7.13 (1H, d, *J* = 2.0 Hz, H-8), 6.99 (1H, s, H-3), 6.86 (1H, d, *J* = 2.0 Hz, H-6), 5.77 (1H, d, *J* = 6.0 Hz, H-7''), 5.76 (1H, d, *J* = 7.2 Hz, H-1'''), 4.95 (1H, q, H-8''), 4.54 (1H, br. d, *J* = 10.4 Hz, H-6'''a), 4.43 (1H, dd, *J* = 11.6, 4.0 Hz, H-9''a), 4.37–4.10 (5H, m, H-2''', H-3''', H-4''', H-5''', 6'''b-OCH_2_), 4.06 (1H, dd, *J* = 12.0, 4.0 Hz, H-9''b), 3.82 (6H, s, OCH_3_-3', 5'), 3.77 (3H, s, OCH_3_-3''). ^13^C-NMR (pyridine-*d_5_*) δ 183.5 (C-4), 165.0 (C-7), 164.9 (C-2), 163.3 (C-5), 158.6 (C-9), 154.6 (C-3′, 5'), 149.1 (C-3''), 148.2 (C-4''), 141.9 (C-4'), 134.6 (C-1''), 127.2 (C-1'), 121.4 (C-6''), 116.8 (C-5''), 112.6 (C-2''), 107.4 (C-3), 106.6 (C-10), 105.9 (C-2', 6'), 105.7 (C-1'''), 101.3 (C-6), 96.3 (C-8), 87.8 (C-8''), 80.0 (C-3'''), 79.2 (C-5'''), 75.5 (C-4'''), 74.4 (C-7''), 71.9 (C-2'''), 63.9 (C-6'''), 62.5 (C-9''), 57.6 (C-3', 5'-OCH_3_), 57.1 (C-3''-OCH_3_).

*Tricin-4*'*-O-(threo-β-guaiacylglyceryl) ether* *7''-O-β-d-glucopyranose* (**4**). Yellow amorphous powder (CH_3_OH); HRESI/MS *m*/*z* 687.1962 [M−H]^−^; IR (KBr) 3406, 1639, 1614, 1495, 1358, 1121 cm^−1^; ^1^H-NMR (pyridine-*d_5_*) and ^13^C-NMR (pyridine-*d_5_*), see Table 1.

*Tricin-4*'*-O-(erythro-β-guaiacylglyceryl) ether* *7''-O-β-d-glucopyranose* (**5**). Yellow amorphous powder (CH_3_OH); HRESI/MS *m*/*z* 687.1952 [M−H]^−^; IR (KBr) 3432, 2502, 1685, 1510, 1420, 1399, 1112 cm^−1^; ^1^H-NMR (pyridine-*d_5_*) and ^13^C-NMR (pyridine-*d_5_*), see Table 1.

**Table 1:** ^1^H- (400 MHz) and ^13^C-NMR (100 MHz) data of compound **4**–**5** (in pyridine-*d_5_*, δ in ppm, *J* in Hz).

No.	Compound 4		Compound 5	
	δ_C_	δ_H,_ Coupling Pattern, *J* in Hz	δ_C_	δ_H_, Coupling Pattern, *J* in Hz
2	165.0		165.2	
3	107.0	6.97, s	107.2	7.00, s
4	183.9		183.9	
5	164.3		164.4	
6	101.3	6.72, d, 2.0	101.4	6.74, d, 2.0
7	167.3		167.3	
8	96.3	6.83, d, 2.0	96.3	6.86, d, 2.0
9	159.7		159.8	
10	106.3		106.3	
1'	127.9		128.2	
2'	105.9	7.27, br. s	106.0	7.29, br. s
3'	155.1		155.1	
4'	141.3		142.0	
5'	155.1		155.1	
6'	105.9	7.27, br. s	106.0	7.29, br. s
1''	133.1		132.1	
2''	114.0	7.50, d, 1.6	113.9	7.59, d, 1.6
3''	149.4		149.4	
4''	148.8		148.9	
5''	117.0	7.18, d, 8.0	117.1	7.21, d, 8.0
6''	122.8	7.37, dd, 8.0, 1.6	122.5	7.43, dd, 8.0, 1.6
7''	82.6	5.89, d, 6.0	81.9	6.02, d, 4.8
8''	87.8	5.19, m	87.6	5.16, m
9''a	62.7	4.75, dd, 12.4, 3.6	63.0	4.44, dd, 12.0, 4.4
9''b		4.36, dd, 12.0, 2.8		4.02, dd, 11.6, 5.2
3'-OCH_3_	57.2	3.81, s	57.7	3.83, s
5'-OCH_3_	57.2	3.81, s	57.7	3.83, s
3''-OCH_3_	56.6	3.73, s	57.1	3.67, s
1'''	105.7	5.54, d, 8.0	105.6	5.35, d, 8.0
2'''	75.5	4.28–4.12, m	77.1	4.31–3.09, m
3'''	79.7		80.1	
4'''	71.9		72.8	
5'''	79.6		79.7	
6'''a	63.0	4.41, m	63.8	4.45, m
6'''b		4.20, m		4.30, m

## 4. Conclusions

Five compounds were isolated from the aerial parts of *Zizania latifolia* using a prep-LC instrument equipped with cartridges of various sizes and were identified based on spectroscopic data analysis, including NMR, UV, and ESI-MS. Four flavonolignan glycosides, including two new flavonolignan glycosides, showed structural similarities in being derived from tricin-7-*O*-β-d-glucopyranose (**1**), and were shown to be diastereomers of each other. Tricin-4'-*O*-(*threo*-β-guaiacylglyceryl) ether 7-*O*-β-d-glucopyranose (**2**) and tricin-4'-*O*-(*erythro*-β-guaiacylglyceryl) ether 7-*O*-β-d-glucopyranose (**3**) were shown to be diastereomers distinguished by *threo* and *erythro* configurations, whereas tricin-4'-*O*-(*threo*-β-guaiacylglyceryl) ether 7''-*O*-β-d-glucopyranose (**4**) and tricin-4'-*O*-(*erythro*-β-guaiacylglyceryl) ether 7''-*O*-β-d-glucopyranose (**5**) were shown to be *threo* and *erythro*, respectively.

## Supplementary Materials

^1^H-NMR, ^13^C-NMR, and HR-ESIMS spectra of **4**–**5** are available as supporting data. Supplementary materials can be accessed at: http://www.mdpi.com/1420-3049/20/04/5616/s1.
