# Supplementary material for: New Flavonolignan Glycosides from the Aerial Parts of Zizania latifolia
Source: Molecules. 2015 Mar 30;20(4):5616–24. doi: 10.3390/molecules20045616 (PMC6272199; doi:10.3390/molecules20045616)
Supplement: Supplementary file 1 [file molecules-20-05616-s001.pdf]

Supporting Information

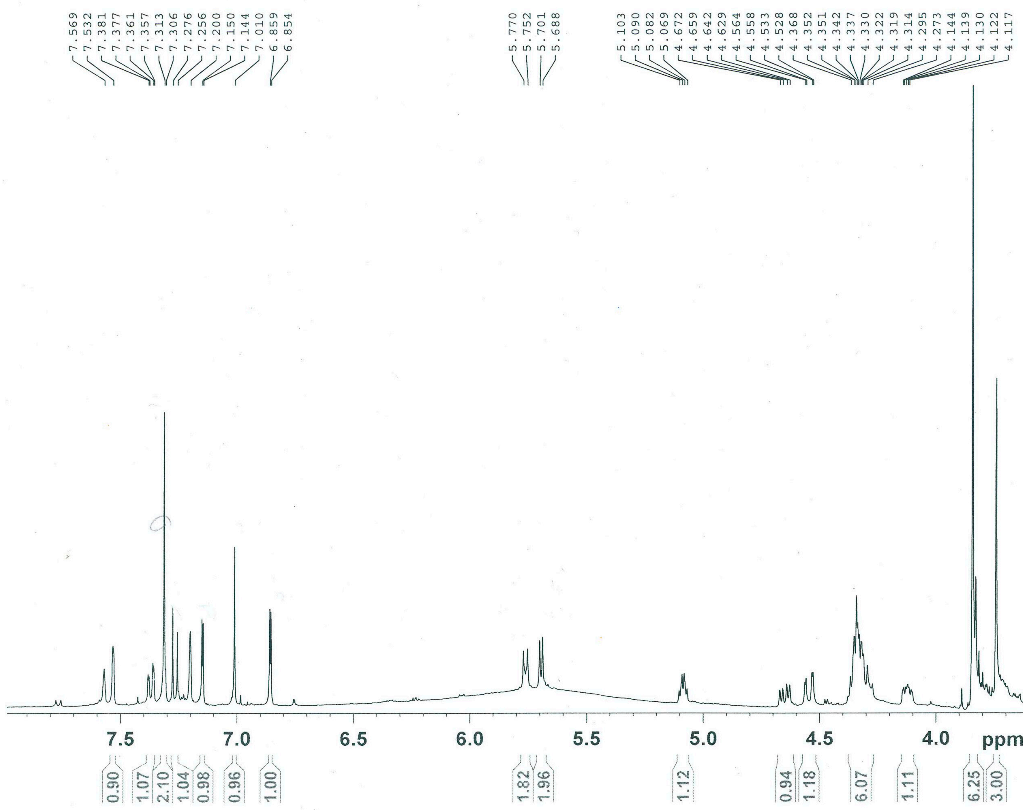

Figure S1. ^{1}H-NMR of compound **4**.

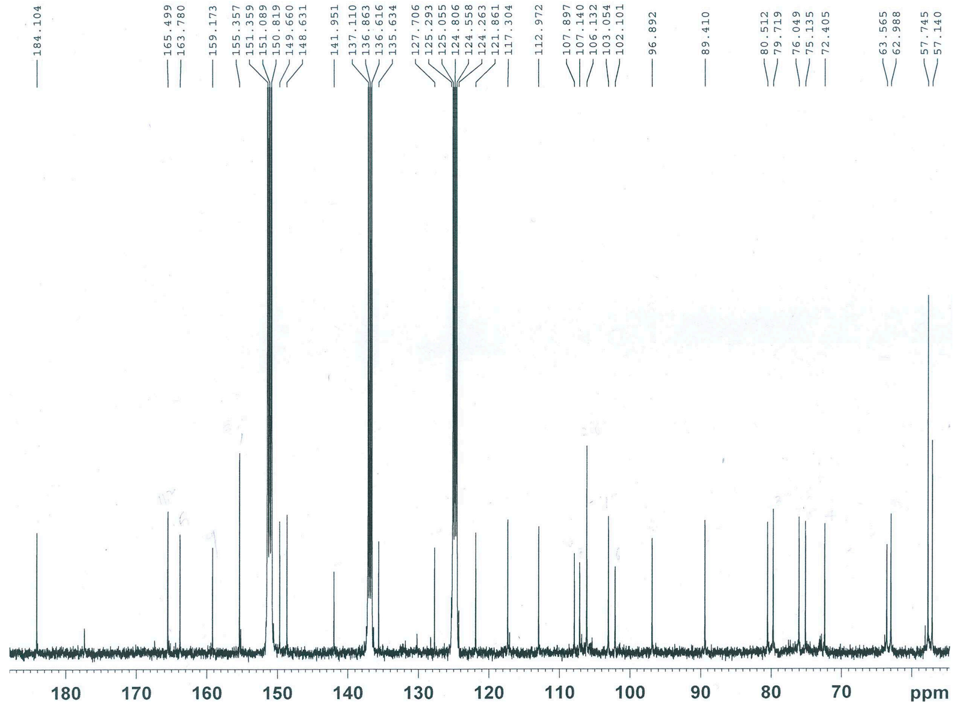

Figure S2. ^{13}C-NMR of compound **4**.

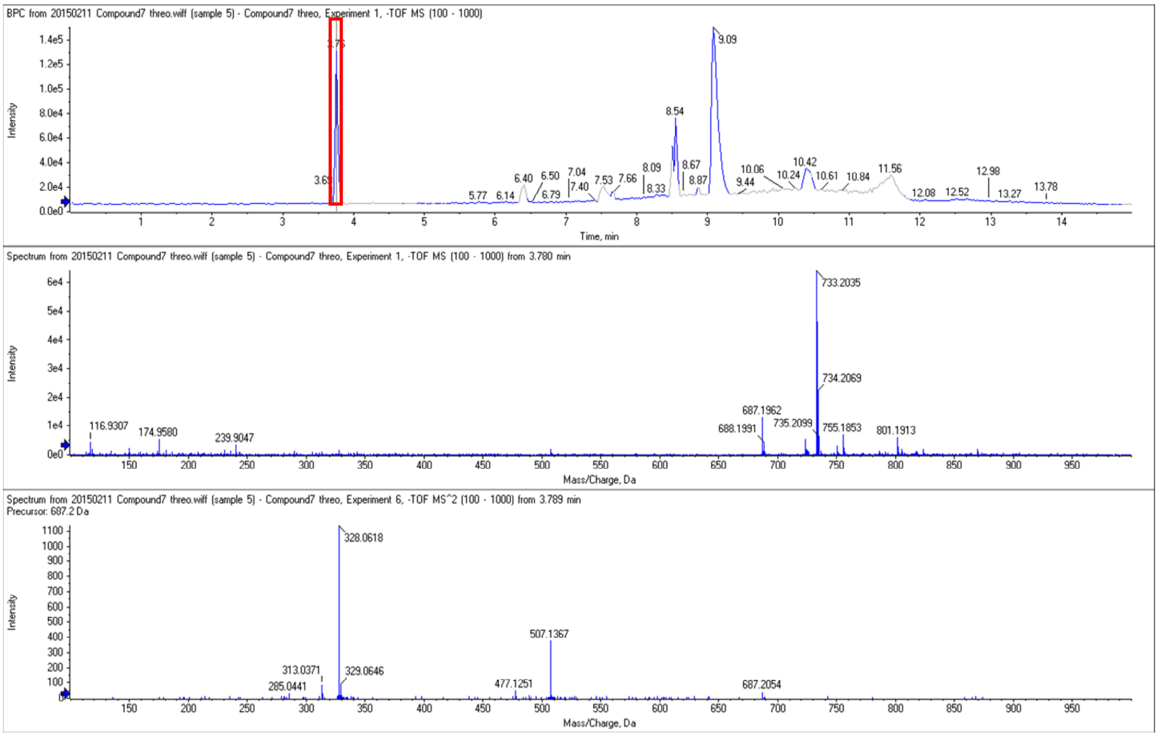

Figure S3. HR-ESIMS of compound **4**.

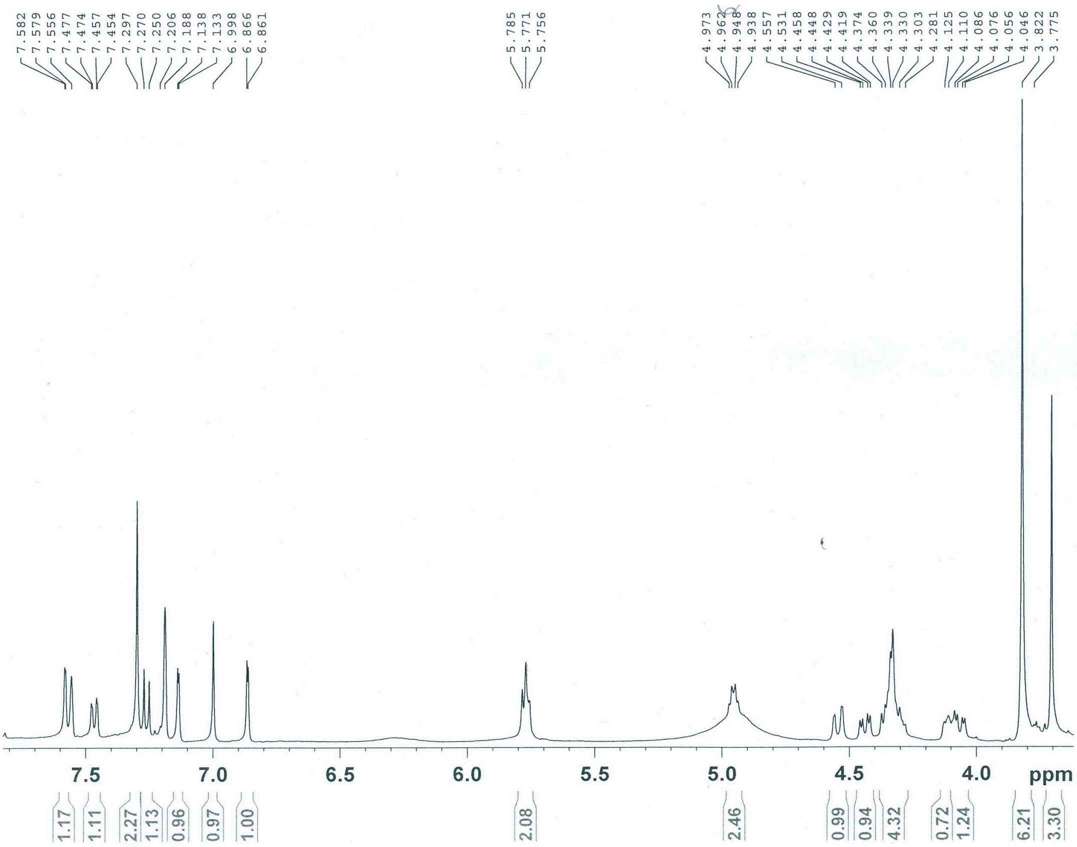

Figure S4. ^{1}H-NMR of compound **5**.

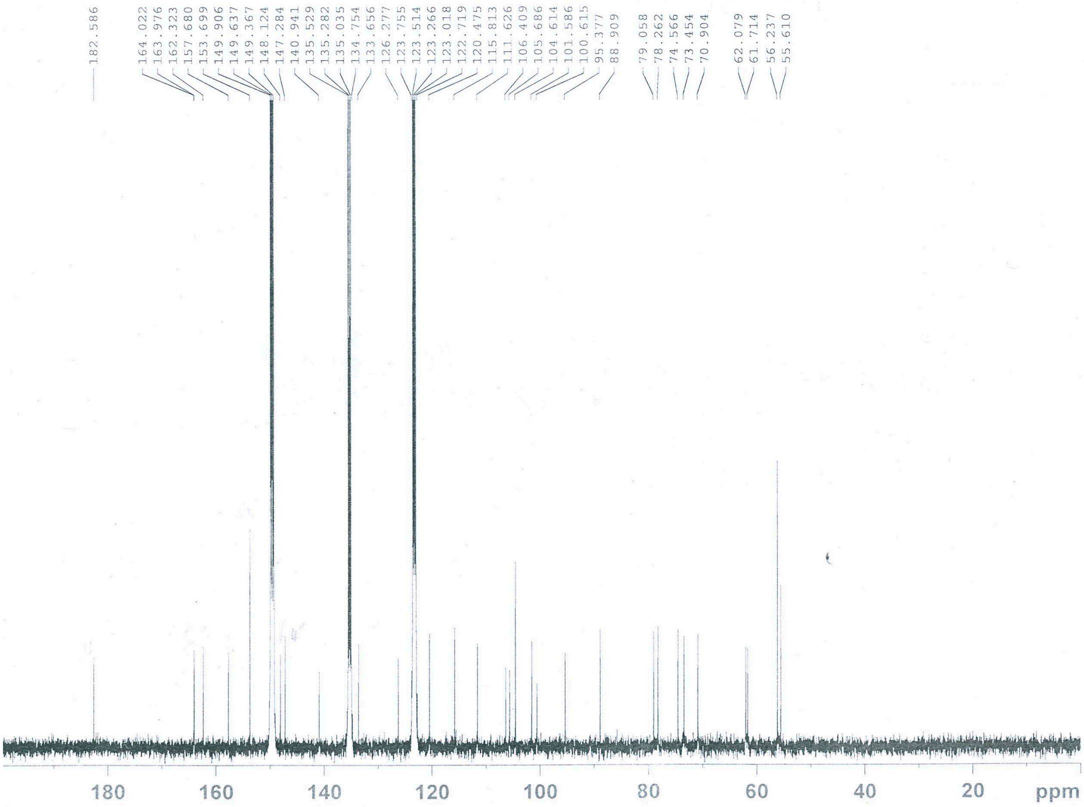

Figure S5. ^{13}C-NMR of compound **5**.

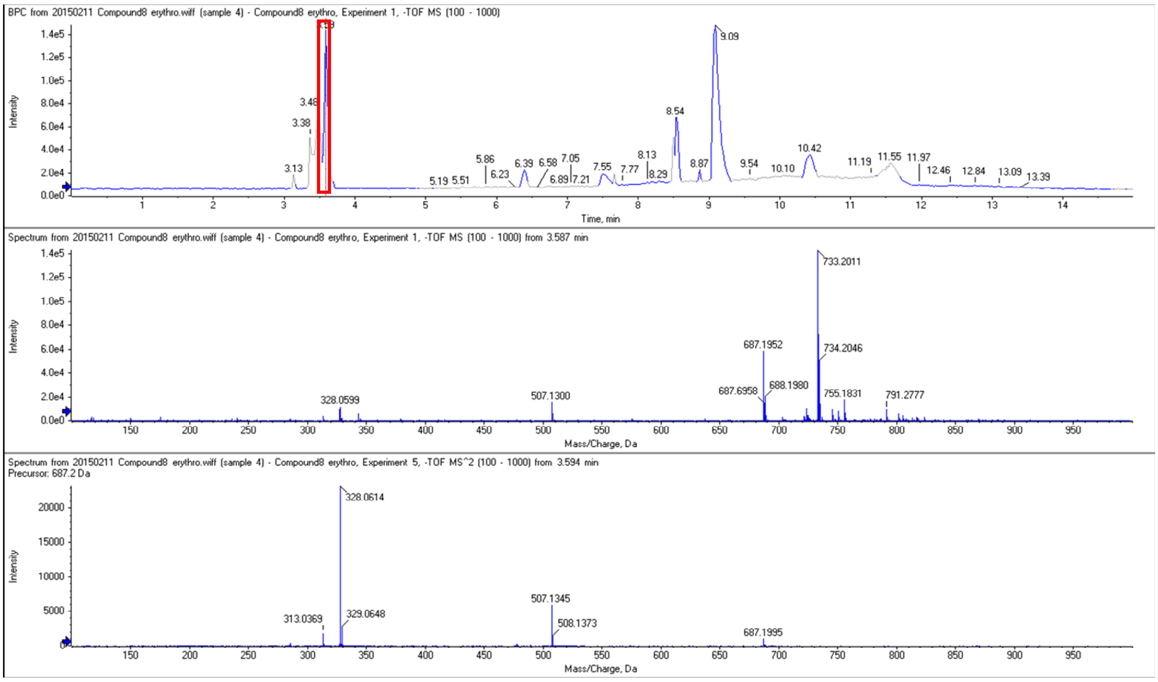

Figure S6. HR-ESIMS of compound **5**.
